# Quantitative LC–MS/MS uncovers the regulatory role of autophagy in immune thrombocytopenia

**DOI:** 10.1186/s12935-021-02249-4

**Published:** 2021-10-18

**Authors:** Rui-Jie Sun, Dong-mei Yin, Dai Yuan, Shu-yan Liu, Jing-jing Zhu, Ning-ning Shan

**Affiliations:** 1 Department of Hematology, Shandong Provincial Hospital, Cheeloo College of Medicine, Shandong University, Jinan, 250021 Shandong China; 2 Department of Blood Transfusion, Shandong Provincial Hospital, Cheeloo College of Medicine, Shandong University, Jinan, 250021 Shandong China; 3 Department of Hematology, Shandong Provincial Hospital Affiliated to Shandong First Medical University, 324 Jing Wu Rd, Jinan, 250021 Shandong China

**Keywords:** LC–MS/MS, Immune thrombocytopenia, Autophagy

## Abstract

**Background:**

Immune thrombocytopenia (ITP) is an autoimmune haemorrhagic disease whose pathogenesis is associated with bone marrow megakaryocyte maturation disorder and destruction of the haematopoietic stem cell microenvironment.

**Methods:**

In this study, we report the qualitative and quantitative profiles of the ITP proteome. Liquid chromatography–tandem mass spectrometry (LC–MS/MS) was conducted to elucidate the protein profiles of clinical bone marrow mononuclear cell (BMMC) samples from ITP patients and healthy donors (controls). Gene Ontology (GO) and Kyoto Encyclopaedia Genes and Genome (KEGG) pathway analyses were performed to annotate the differentially expressed proteins. A protein–protein interaction (PPI) network was constructed with the BLAST online database. Target proteins associated with autophagy were quantitatively identified by parallel reaction monitoring (PRM) analysis.

**Results:**

Our approaches showed that the differentially expressed autophagy-related proteins, namely, HSPA8, PARK7, YWHAH, ITGB3 and CSF1R, were changed the most. The protein expression of CSF1R in ITP patients was higher than that in controls, while other autophagy-related proteins were expressed at lower levels in ITP patients than in controls.

**Conclusion:**

Bioinformatics analysis indicated that disruption of the autophagy pathway is a potential pathological mechanism of ITP. These results can provide a new direction for exploring the molecular mechanism of ITP.

**Supplementary Information:**

The online version contains supplementary material available at 10.1186/s12935-021-02249-4.

## Introduction

Immune thrombocytopenia (ITP) is a multifactorial bleeding disease characterized by a breakdown of immune tolerance leading to a platelet count decline. Bone marrow fluid is a vital bodily tissue that has been extensively studied to elucidate the physiology and pathology of the haematological system. Characteristic pathological changes in megakaryocytes suggest an important role of bone marrow in ITP. Blocking the maturation of megakaryocytes [1, 2] and variations in the bone marrow microenvironment [3] are important contributors to platelet destruction and/or suppression of platelet production in subjects with ITP [4].

Autophagy, also called self-feeding, is a highly conserved catabolic process in eukaryotic cells. Autophagy is involved in cell development, intracellular quality control, adaptation, starvation, ageing, tumour suppression, innate immunity, and other processes [5]. Autophagy also plays an important role in maintaining the microenvironment and stemness of haematopoietic stem cells [5] and in regulating megakaryopoiesis and platelet function [6]. Wang et al. reported that autophagy was inhibited by bafilomycin A1 or induced by rapamycin in bone marrow cells and observed a significant decrease in high-ploidy megakaryocytes and reductions in CD41 and CD61 (two megakaryocytic cell markers) coexpressing cells, proplatelets and platelet formation [7]. Our previous study performed using the human Dami cell line confirmed that autophagy is involved in megakaryocyte endomitosis and platelet development in vitro [8]. As emerging roles of abnormal autophagy in megakaryopoiesis, thrombopoiesis and platelet function have been revealed in ITP patients, insights into signalling pathways may guide future research in this field.

As a powerful technique for biomarker discovery, proteomic strategies have been utilized to study many haematological system malignant diseases, such as acute myelogenous leukaemia (AML) [9] and myelodysplastic syndrome (MDS) [10], revealing the great potential of bone marrow in biomarker discovery and clinical tests. However, bone marrow mononuclear cell (BMMC) protein expression in the context of ITP has not been analysed, which is necessary for deeper pathogenesis research.

In this study, we established an integrated workflow based on the combination of proteome quantification and PRM validation of bone marrow from ITP patients and healthy controls. With the help of advanced bioinformatics, we hoped to further our understanding of the pathogenesis and provide a novel therapeutic approach for ITP. We performed Gene Ontology (GO), Kyoto Encyclopaedia Genes and Genome (KEGG), protein domain enrichment and clustering analyses to determine the correlations between the functions and differential expression of proteins. PRM analysis further confirmed that the expression of autophagy-related proteins (HSPA8, PARK7, YWHAH, ITGB3 and CSF1R) was significantly different in ITP patients and healthy controls. In addition, we indicated that the five autophagy-related differentially expressed proteins were closely related to mammalian target of rapamycin (mTOR) signalling or similar pathways (such as mitogen-activated protein kinase (MAPK) to thereby regulate autophagy activity, and these results may provide useful diagnostic and targeted treatment information for ITP patients in the future.

## Materials and methods

### Patient and control samples

The ethics protocol for the collection of human bone marrow aspirate with informed consent was approved by Shandong Provincial Hospital affiliated with Shandong University and Shandong Provincial Hospital affiliated with Shandong First Medical University (ethics approval number: No. 2021-292). Twenty newly diagnosed primary ITP patients in the active phase (12 females and 8 males, age range of 18–70 years, median of 42 years) and 20 healthy controls (12 females and 8 males, age range of 18–55 years, median of 46 years) were enrolled in this study between May and November 2018. All research participants, including the patients and healthy controls (donors), provided informed consent. The diagnosis of ITP was made according to recently published criteria, including patient history, complete blood count, physical examination and peripheral blood smear examination [11]. The patients' platelet counts ranged from 1 to 30 × 10^9^/l, with a median platelet count of 11 × 10^9^/l (Table 1) [12]. Patients and controls were divided into four groups according to their age and sex to reduce differences and increase the accuracy of the results. None of the subjects had been treated with glucocorticosteroids, immunoglobulins or immunosuppressants prior to sampling. Bone marrow aspirate was collected into heparin-containing vacutainer tubes. According to the manufacturer’s instructions, BMMCs were isolated from heparinized bone marrow aspirate samples by Ficoll-Paque gradient centrifugation (Pharmacia Diagnostics, Uppsala, Sweden). BMMCs from ITP and control subjects were stored at − 80 °C.

**Table 1:** Clinical characteristics of ITP patients

Group	Group 1	Group 2	Group 3	Group 4	Median (min.–max.)
Age(year)/sex	19/F	18/F	18/F	43/F	42 (18–70)
	38/F	27/M	33/M	69/F	
	43/F	41/F	48/F	48/F	
	55/M	45/M	43/F	25/M	
	70/M	65/F	60/M	39/M	
Platelet counts (× 10^9^/l)	9	30	11	1	11 (1–30)
	7	18	11	8	
	12	17	12	3	
	3	8	4	12	
	14	29	4	14	
Bleeding symptoms	EC, PT	EC	PT	PT, GH	
	GUH, PT	NONE	GH	PT, GUH	
	GH, EP	GUH, PT	GH	PT, GH	
	GH	GH, EC	EP, GH	GH, EP	
	NONE	EP, GH	EC, GH	GH	

*EC* ecchymoses, *PT* petechiae, *GUH* genitourinary hemorrhage, *GH* gingival hemorrhage, *EP* epistaxis

### Crude protein extraction and trypsin digestion

The samples were centrifuged at 12,000*g* for 10 min at 4 °C, and the cell debris was discarded. After transferring the supernatant to a new centrifuge tube, the protein was precipitated with 20% cold trichloroacetic acid (TCA) and washed with cold acetone. An Abundant Protein Depletion Kit (Pierce Top 12, Thermo) was used to remove the highly abundant proteins. The proteins were redissolved in buffer (8 M urea, 100 mM TEAB, pH 8.0), and the protein concentration was determined with a BCA kit. The protein solution was digested with 5 mM dithiothreitol (Sigma) at 56 °C for 30 min and then alkylated with 11 mM iodoacetamide (Sigma) in the dark at room temperature for 15 min. The protein sample was then diluted with 100 mM TEAB to decrease the urea concentration to less than 2 M. Finally, trypsin was added at a 1:50 trypsin-to-protein mass ratio for the first digestion overnight and at a 1:100 trypsin-to-protein mass ratio for a second 4 h digestion to improve the digestion effect [13, 14].

### High-performance liquid chromatography (HPLC) fractionation and LC MS/MS analysis

Tryptic peptides were fractionated by high pH reverse-phase HPLC on an Agilent 300Extend C18 column (5 μm particles, 4.6 mm ID, 250 mm length). Peptides were separated into 60 fractions with an acetonitrile (pH 9.0) gradient of 8–32% over 60 min. They were then combined into 4 fractions and dried by vacuum centrifugation. The peptides were redissolved in solvent A (0.1% formic acid in 2% acetonitrile) and loaded onto a reverse-phase analytical pre-column (Acclaim PepMap 100, Thermo Scientific). The gradient was as follows: 6–25% solvent B (0.1% formic acid in 90% acetonitrile) over 40 min; 25–35% over 12 min; 35–80% over 4 min; and holding at 80% for 4 min. An EASY-nLC 1000 UPLC system was utilized at a constant flow rate of 500 nL/min. The peptides were subjected to a nanospray ionization (NSI) source on the Q Exactive™ Plus (Thermo) instrument coupled online to the UPLC and detected by the Orbitrap. A data-dependent procedure (DDA) that alternated between one MS scan followed by 20 tandem mass spectrometry (MS/MS) scans was performed. Automatic gain control (AGC) was used to prevent overfilling of the Orbitrap, and 5E4 ions were accumulated for the generation of MS/MS spectra; the maximum injection time was set at 30 ms, and the signal threshold was set at 15,000 ions/s.

### Bioinformatics/functional enrichment analyses

The resulting MS/MS data were processed using the Maxquent search engine (v.1.5.2.8).

InterProScan software was used to identify protein domain functions based on the protein sequence alignment method. Then, proteins were classified by GO annotation based on three categories: biological process, cellular component and molecular function. The KEGG online service tool KAAS was used to annotate the protein KEGG database descriptions. The annotation results were then mapped to the KEGG pathway database using KEGG mapper. The GO and KEGG analysis results were subjected to two-tailed Fisher’s exact tests to determine the enrichment of the differentially expressed proteins against all identified proteins. A corrected p-value < 0.05 was considered significant. All categories, and their p values, were collated after enrichment, and those that were enriched in at least one of the clusters and had a p value < 0.05 were filtered. The clusters were visualized as a heat map using the “heatmap.2” function. All differentially expressed proteins were searched against the STRING database version 10.1 for protein–protein interactions (PPIs) and visualized with the R package “networkD3”.

### Parallel reaction monitoring (PRM) analysis

Target proteins associated with autophagy were quantitatively identified using mass spectrometry-based targeted proteome quantification. The proteins selected for PRM were based on the quantitative analysis of the bone marrow serum proteome. The crude protein extraction and trypsin digestion steps were similar to those described above. The electrospray voltage was 2.0 kV, and the peptide length was set to 7–25. The transition settings were as follows: precursor charges were set to 2 and 3, ion charges were set to 1, and ion types were set to b and y. The product ions were set from ion 3 to the last ion, and the ion match tolerance was set to 0.02 Da. The PRM data were analysed using Skyline (v.3.6) software.

### Statistical analysis

Statistical analyses were performed using GraphPad Prism 8 software. For PRM data analysis, two groups were compared by two-tailed Student’s t-tests. A p-value < 0.05 was considered statistically significant.

## Results

### Proteome quantification overview

**Fig. 1:**
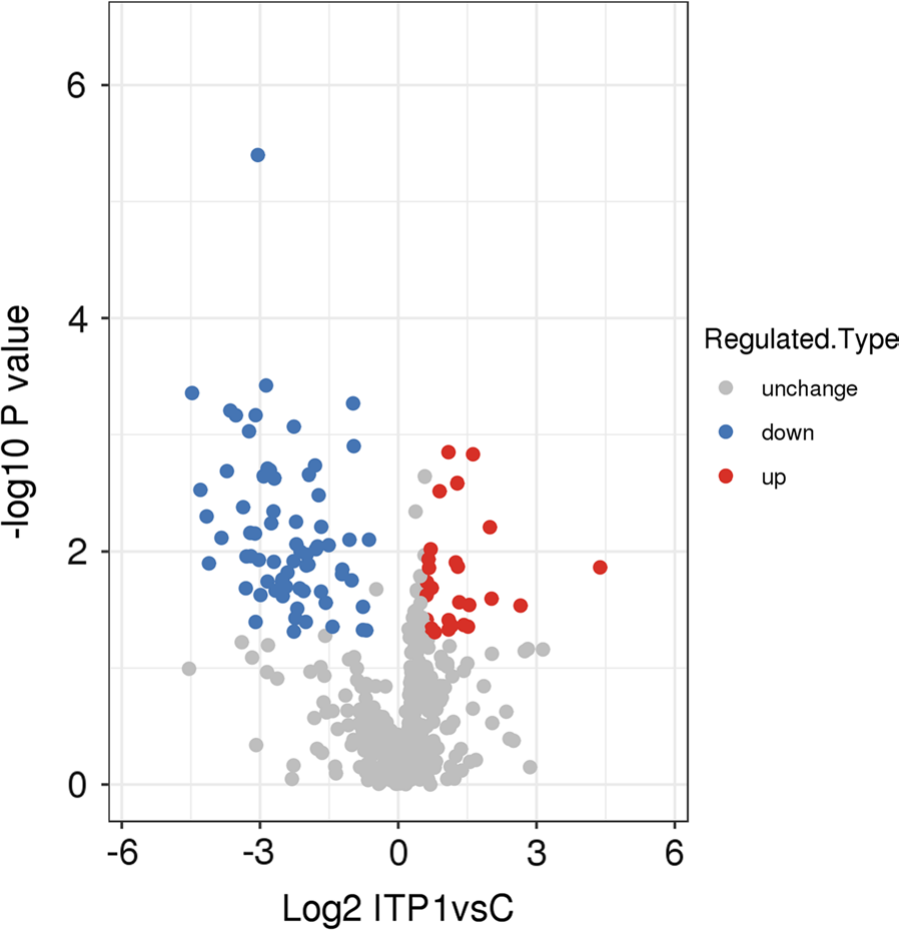
Volcano plots of all proteins identified by LC–MS/MS analysis. The red dots in these plots represent the upregulated proteins with statistical significance, and the blue dots represent the downregulated proteins (fold change ≥ 1.5, p ≤ 0.05)

In this study, we established an integrated method for quantifying the proteome of human BMMCs. We assessed the data accuracy and validated the sample preparations meeting the requirements; we identified 829 proteins in human BMMCs, among which 613 proteins were quantified. In total, 69 proteins were downregulated with a fold change < 1/1.5, while 26 proteins were upregulated with a fold change > 1.5 (p ≤ 0.05). To identify differentially expressed proteins, volcano plot analysis was conducted to visualize the differences between the ITP patient and control groups (Fig. 1).

### Abnormal regulation of autophagy-related proteins (HSPA8, PARK7, YWHAH, ITGB3, and CSF1R)

**Fig. 2:**
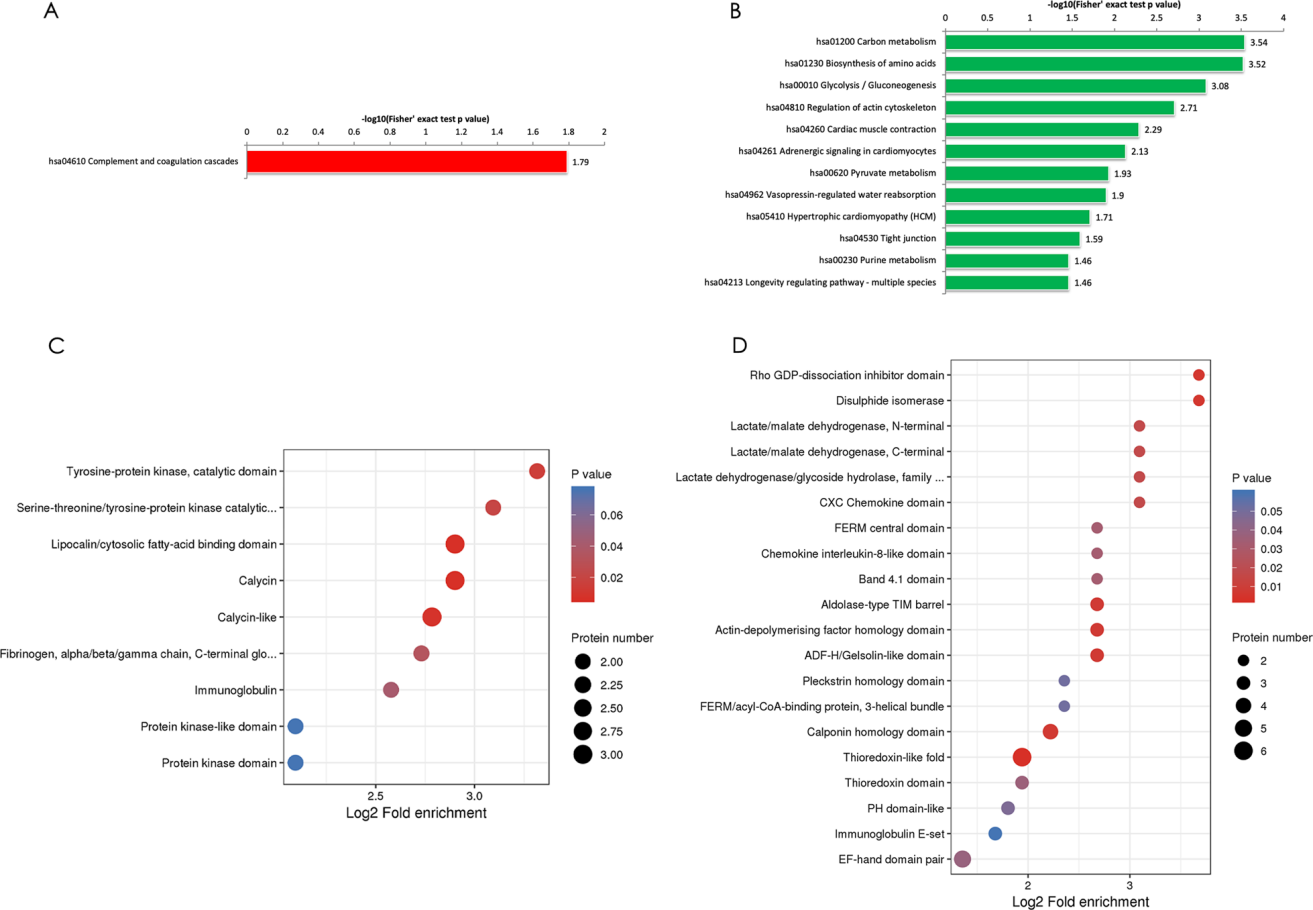
Functional enrichment of differentially expressed proteins. **A** KEGG enrichment analysis of the upregulated differentially expressed proteins as determined by Fisher’s exact test p values (− log10). **B** KEGG enrichment analysis of the downregulated differentially expressed proteins as determined by Fisher’s exact test p values (− log10). **C** Protein domain enrichment analysis of the upregulated proteins. The results of the domain enrichment analysis of differentially expressed proteins are displayed as a bubble chart. **D** Protein domain enrichment analysis of the downregulated proteins. The circle size indicates the number of differentially expressed proteins, and the circle colour indicates the enrichment significance of the p value

To identify the functional classifications and pathways of the differentially expressed proteins in ITP, we performed KEGG pathway enrichment analysis. Our data showed that the upregulated differentially expressed proteins in ITP were most prominently enriched in the complement and coagulation cascades and that the downregulated differentially expressed proteins in ITP were enriched in the carbon metabolism (Fig. 2A), regulation of actin cytoskeleton, vasopressin-regulated water reabsorption and tight junction pathways (Fig. 2B). Using the networkD3 R package, we revealed some highly connected subnetworks among autophagy proteins, including heat shock protein family A (Hsp70) member 8 (HSPA8), Parkinson’s disease (autosomal recessive, early onset) 7 (PARK7), tyrosine 3-monooxygenase/tryptophan 5-monooxygenase activation protein, eta polypeptide, isoform CRA_b (YWHAH), integrin beta-3 (ITGB3) and colony-stimulating factor 1 receptor (CSF1R). The expression of the CSF1R protein in ITP patients was higher than that in controls, while the other autophagy-related proteins were expressed at lower levels in ITP patients than in controls.

The downregulated protein HSPA8 participates in signalling pathways associated with the longevity of multiple species and endocytosis (Additional file 1: Table S1). YWHAH was downregulated and enriched in the cell cycle, vasopressin-regulated water reabsorption and Hippo signalling pathways, and ITGB3 was downregulated and enriched in the focal adhesion, platelet activation and haematopoietic cell lineage pathways (Additional file 1: Table S1). The autophagy-related CSF1R protein was upregulated and closely related to the haematopoietic cell lineage and cytokine-cytokine receptor interactions (Additional file 1: Table S1).

Protein functions are largely dependent on specific domain structures in their sequence. To assess the domain structures, a bubble chart was constructed by Fisher’s exact test [log2(p value)] to analyse protein domain enrichment. In agreement with our findings, the protein domains associated with lipocalin/cytosolic fatty-acid binding and calycin were enriched in the upregulated proteins (Fig. 2C) in ITP, while the thioredoxin-like fold was the most enriched domain among the downregulated proteins (Fig. 2D). CSF1R, an autophagy-related protein, was mainly related to the immunoglobulin-like domain, serine-threonine/tyrosine-protein kinase catalytic domain and tyrosine-protein kinase catalytic domain (Additional file 2: Table S2). Some important domains of other downregulated autophagy proteins were enriched, such as the 14-3-3 domain of YWHAH and the C-terminal domain of HSPA8 (Additional file 2: Table S2).

### Clustering analysis

**Fig. 3:**
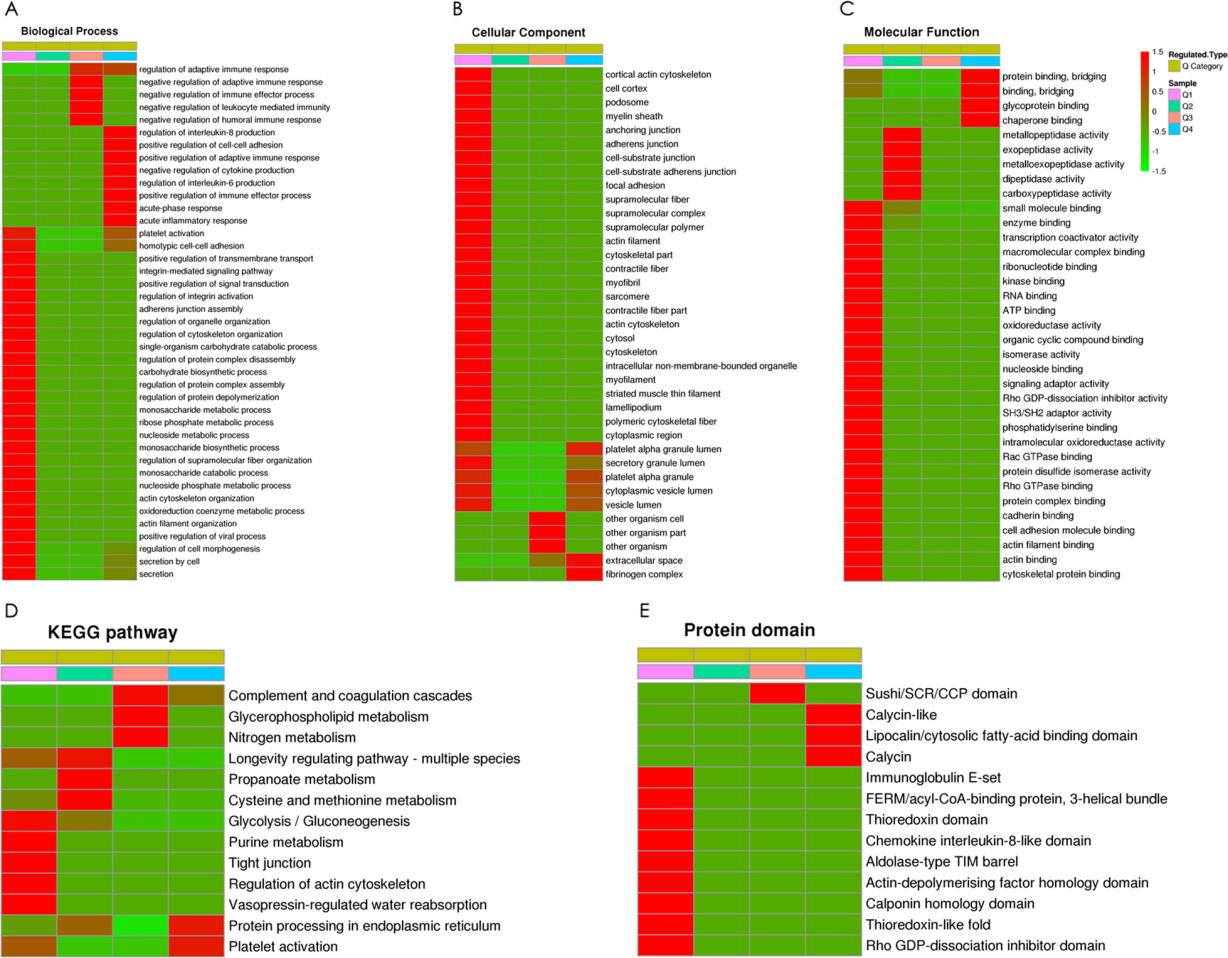
Heatmaps of clusters based on the enrichment of (**A**) GO biological processes, (**B**) GO cellular components, (**C**) GO molecular functions, (**D**) KEGG pathways, and (**E**) protein domains. The differentially expressed proteins were divided into four quantitative categories according to the ITP patient group (P) to control group (C) ratio: Q1 (0 < P/C ratio < 1/2, p value < 0.05) and Q2 (1/2 < P/C ratio < 1/1.5, p value < 0.05) represent downregulated proteins, and Q3 (1.5 < P/C ratio < 2, p value < 0.05) and Q4 (P/C > 2, p value < 0.05) represent upregulated proteins

To assess the correlations between the functions of differentially expressed proteins in the ITP patient group, all the differentially expressed proteins were divided into four quantiles (Q1–Q4) according to their expression ratios as described above. Then, enrichment-based clustering analyses (GO, KEGG and protein domain) were performed (Fig. 3).

The biological process category was analysed as shown in Fig. 3A. The downregulated proteins were highly enriched in the platelet activation, cell−cell adhesion, and integrin-mediated signalling (Q1) terms, which may be associated with the autophagy process. The upregulated proteins were highly enriched in the negative regulation of adaptive immune response term in Q3 and in the acute inflammatory response, positive regulation of adaptive immune response and negative regulation of cytokine production terms in Q4, which may be attributed to the mechanism underlying the unbalanced autoimmune response in ITP. In the cellular component category, the upregulated proteins were mainly located in the organism cell part (Q3) and in the extracellular space and fibrinogen complex (Q4), while the downregulated proteins were mainly located in the cytoplasmic region (Fig. 3B). The results of molecular function analysis are presented in Fig. 3C. Downregulated proteins with small molecule binding activity, signalling adaptor activity and transcription coactivation activity were enriched in Q1. The chaperone binding function of upregulated proteins was enriched in Q4.

**Fig. 4:**
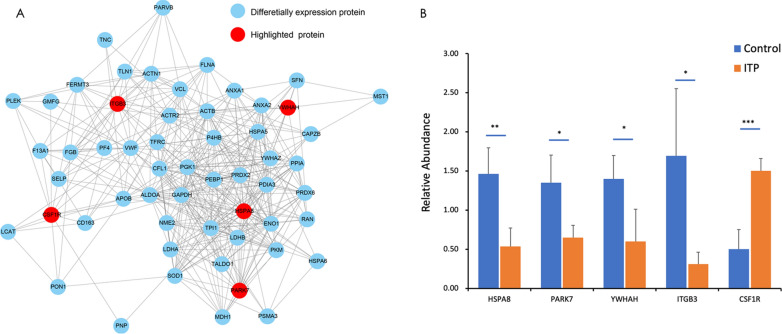
PPI network and PRM analysis of autophagy-related proteins. **A** A complete PPI network was constructed. Five autophagy-related proteins (HSPA8, PARK7, YWHAH, ITGB3 and CSF1R) were highlighted among the differentially expressed proteins. **B** The quantitative analysis results of autophagy-related proteins (HSPA8, PARK7, YWHAH, ITGB3 and CSF1R) were verified by PRM. With the exception of CSF1R, these autophagy-related proteins was significantly downregulated in the ITP group compared with the control group. The data are presented as the mean ± SD; two-tailed Student’s t-test, *p < 0.05, **p < 0.01, ***p < 0.001

KEGG pathway analysis of the differentially expressed proteins in ITP revealed several vital pathways (Fig. 4D). The tight junction pathway was enriched in Q1. The upregulated proteins were associated with the platelet activation pathway in Q4. CSF1R, YWHAH and ITGB3 were enriched in the PI3K/Akt/mTOR signalling pathway (Additional file 3: Table S3). In addition, CSF1R and HSPA8 were shown to participate in the MAPK signalling pathway, which plays crucial roles in various antiproliferative events, including apoptosis and autophagy [15]. Therefore, autophagy-related proteins (HSPA8, CSF1R, YWHAH and ITGB3) were shown to be involved in the pathogenesis of ITP by affecting autophagy and its related pathways.

Domain enrichment analysis of differentially expressed proteins (Fig. 3E) showed that the downregulated proteins clustered with the immunoglobulin E-set, thioredoxin domain and chemokine interleukin-8-like domain, while the upregulated proteins clustered with the fibrinogen, alpha/beta/gamma chain and calycin-like lipocalin/cytosolic fatty-acid binding domains.

### PPI network

We visualized a PPI network of all quantified proteins using the Search Tool for the Retrieval of Interacting Genes/Proteins (STRING) (V.10.5) database. A complete network of differentially expressed proteins was created and included 50 interactions. We found that autophagy-related proteins were clearly upregulated (CSF1R) in the ITP patient group, while a number of proteins (HSPA8, PARK7, YWHAH, and ITGB3) related to autophagy were downregulated. Our data set offers insights into the probability of interactions among autophagy proteins in ITP. A representative example is shown in Fig. 4A.

### PRM analysis

PRM quantification was carried out for 20 selected target proteins in all samples. Limited by the characteristics of some proteins and the abundance of their expression, we quantified 14 of these selected target proteins. The peak area was used for PRM quantitative analysis. The PRM quantitative results for autophagy-related proteins, shown in Fig. 4B, further verified that HSPA8, PARK7, YWHAH, and ITGB3 were downregulated and that CSF1R was upregulated in the ITP patient group compared with the control group.

## Discussion

Proteomics has the potential to provide clues to cancer and autoimmune disease pathogeneses based on comprehensive analyses of protein expression and activation statuses. In our study, we employed a quantitative proteomics strategy to compare the autophagy-related proteins that were differentially expressed in the BMMCs of ITP patients and control subjects and to explore the potential mechanism of ITP pathogenesis. The data showed that 26 upregulated proteins in ITP were enriched in the acute-phase response and regulation of the adaptive immune response, while 69 downregulated proteins were enriched in processes associated with binding, such as actin binding and cytoskeletal protein binding, which manifested as changes in the autophagy fractions. Then, we detected 5 abnormally expressed autophagy-related proteins that may be associated with the pathogenesis of ITP, among which 4 (HSPA8, PARK7, YWHAH, and ITGB3) were downregulated and 1 (CSF1R) was significantly upregulated in ITP patients compared with the controls. Clustering analysis showed that most of the autophagy-related differentially expressed proteins (YWHAH, ITGB3 and CSF1R) identified herein were closely related to the PI3K/Akt/mTOR signalling pathway. The mTOR signalling pathway mediates many physiological functions, such as cell proliferation, differentiation, migration and apoptosis, and regulates autophagy [16, 17]. Studies have shown that the pathway controlling mTOR expression negatively regulates autophagy in cells stimulated by factors such as starvation and hypoxia [18].

YWHAH is a large family of phosphoregulatory proteins that exist primarily as homo- and heterodimers [19]. YWHAH proteins are involved in different signalling pathways that modulate cellular and whole-body energy as well as nutrient homeostasis, such as the insulin and mTOR- and AMP-dependent kinase (AMPK) signalling pathways, and regulate autophagy [20]. Considerable cross-talk exists between the AMPK pathway and other key energy regulatory pathways, such as insulin signalling and mTOR signalling complex 1 (mTORC1) [20]. AMPK is reported to inhibit mTORC1 by activating tuberous sclerosis proteins 1 and 2 (TSC1/2) and by inhibiting regulatory-associated protein of TOR (RAPTOR) via the phosphorylation-induced binding of YWHAH [21], both of which stimulate autophagy. Recently, a direct stimulatory pathway correlating AMPK with autophagy was described based on the phosphorylation of ULK1 [22], and complex formation among ULK1, mTORC1 and AMPK was found to coincide with the phosphorylation of RAPTOR and the binding of YWHAH [23]. We speculated that abnormal autophagy is associated with ITP in patients with low YWHAH protein expression, as it inhibits RAPTOR, ultimately decreasing the function and quantity of megakaryocytes and platelets and leading to the onset of ITP.

KEGG enrichment analysis of the differentially expressed proteins showed that the downregulated autophagy-related protein ITGB3 was also enriched in platelet activation and haematopoietic cell lineages. ITGB3 is an important molecule involved in cell survival, proliferation and cancer metastasis [24]. ITGB3 has been reported to be upstream of the PI3K/AKT/mTOR signalling pathway in various cell types, and the pathway is activated when ITGB3 is overexpressed [25]. Studies have shown that ITGB3 upregulation inhibits the autophagic process in cardiomyocytes by activating AKT, suggesting that the expression status of ITGB3 affects cell autophagy [26]. ITGB3 encodes GP3A (also known as GPIIIa) and represents a common platelet antigen polymorphism (PIA1/A2) that can influence platelet activation and aggregation. ITGB3 can provide instructions for forming the β3 subunit of the receptor integrin αIIbβ3. ITGB3 mutations cause activation of alphaIIb/beta3 (αIIbβ3) and leads to platelet dysfunction and macrothrombocytopenia [27]. Another study found that eltrombopag can induced relevant changes in the hematopoiesis, platelet activation as well as megakaryocyte differentiation with the overexpression of the genes ITGB3 [28].Our results showed that the expression of ITGB3 in the ITP patient group was lower than that in the control group, suggesting that the overexpression of autophagy may be caused by the downregulation of AKT activation by ITGB3.

CSF1R was herein identified as another important autophagy-related protein. Autophagy mediated by CSF-1/CSF1R plays a crucial role during the differentiation of human monocytes into macrophages [29, 30], which induce typical autophagic structures, such as phagophores and autophagosomes, and results in the accumulation of LC3-II [29]. Tian et al. showed that the level of LC3-II was lower in cells overexpressing CSF1R-Mut than in benign control or CSF1R-WT cells exposed to CSF-1 stimulation, indicating that the autophagy process might be disturbed by abnormal CSF-1/CSF1R signalling [31]. At the molecular level, E5[*N*-(3-((4(benzofuran-2-yl) pyrimidin-2-yl) oxy)-4-methylphenyl)-4-((4-methylpiperazin-1-yl) methyl) benzamide] was able to downregulate the mTOR pathway and activate the MAPK/ERK pathway [15, 32], thus inducing the conversion of LC3-I to LC3-II, increasing the expression of Atg5 and restoring autophagy. In our study, the expression level of CSF1R was decreased in the control group compared with the ITP patient group, which may demonstrate that abnormal autophagy mediated by CSF1/CSF1R signalling is involved in the pathogenesis of ITP.

HSPA8 was enriched in the longevity regulating pathway-multiple species as determined by KEGG analysis. HSPA8 is a molecular chaperone involved in a wide variety of cellular processes that localizes in the nucleus, cytosol, extracellular exosomes, and cell membrane [33]. HSPA8 detects substrates that are processed by chaperone-mediated autophagy (CMA) [34], and its expression is altered in a number of immune disorders. For example, flow cytometry studies showed that the expression of HSPA8 was increased in the splenic B and T cells of MRL/MpTn-gld/gld lupus-prone mice [35, 36]. HSPA8 was also shown to be involved in the molecular regulation of haematopoiesis [37]. PARK7 is a multifunctional protein that is involved in various cellular activities, and its principle functions include antioxidative defence and mitochondrial homeostasis maintenance [38]. PARK7 dysfunction leads to mitochondrial defects. Furthermore, CMA protects cells from mitochondrial toxin MPPC-induced changes in mitochondrial morphology and function and increases cell viability [39]. Under PARK7-deficient conditions in subjects with ITP, these protective effects may be lost.

In recent years, experimental and clinical evidence has concluded that autophagy plays an important role in maintaining the stemness and microenvironment of haematopoietic stem cells [5]. Perturbations of normal autophagy processes in ITP patients may be caused by the deletion of autophagy-related genes such as ATG7 and abnormal signalling due to the overexpression of mTOR. These changes are thought to affect markers of haematopoietic stem cells, such as CD41 and CD61, and the differentiation of megakaryocytes, ultimately decreasing the function and quantity of platelets and leading to the onset of ITP [3]. Ouseph et al. demonstrated that the autophagy process is essential for the normal functioning of platelet activation and aggregation [40]. In another study, they demonstrated that starvation induced substantial autophagy (above basal level), which was characterized by decreased platelet aggregation, reduced calcium mobilization and granule secretion, decreased adhesion to immobilized fibrinogen, and eventually, an increased bleeding time [41].

In this study, we indicated five autophagy-related differentially expressed proteins in ITP BMMC samples. GO, KEGG, protein domain enrichment and clustering analyses were performed to determine the correlations between the functions and differential expression of proteins. PRM analysis further confirmed that the expression of autophagy-related proteins was significantly different in ITP patients and health controls. CSF1R in ITP patients was higher than that in controls, while other autophagy-related proteins, HSPA8, PARK7, YWHAH, ITGB3, were expressed at lower levels in ITP patients than in controls. Furthermore, we indicated that the five autophagy-expressed proteins were closely related to mTOR signaling or similar pathways that regulate autophagy activity, and these results may provide more strategies for ITP targeted treatments and diagnosis in the future.

In the follow-up experiments, Western Blot, immunohistochemistry, RT-PCR and other biological techniques will be used to verify the expression levels of five autophagy-related proteins in more specimens. The differential condition of these autophagy-related proteins in the levels of proteins and transcription will be observed in vitro experiments with bafilomycin A1 and rapamycin. The expression level of autophagy differential proteins can be detected after the treatment of in ITP mouse by blocking or enhancing targeted proteins. These will be helpful for further confirmation the relationship between the five autophagy-related proteins (especially YWHAH, CSF1R and ITGB3) and ITP bone marrow megakaryocyte cells and their specific role in PI3K/Akt/mTOR pathway.

## Supplementary Information

**Additional file 1: Table 1**. The description of KEGG pathway.

**Additional file 2: Table 2**. The description of protein domains.

**Additional file 3: Table 3**. The description of clustering analysis.

## Data Availability

All of the data in this manuscript are presented in the main paper.
